# Green synthesis of new and natural diester based on gallic acid and polyethylene glycol

**DOI:** 10.12688/f1000research.139861.1

**Published:** 2023-10-20

**Authors:** Hafida Zerigui, Radia Labied, Redouane Chebout, Khaldoun Bachari, Rachid Meghaber, Fatima zohra Zeggai

**Affiliations:** 1 Laboratoire de chimie des polymères, Université Oran1 Ahmed Benbella, BP 1524, El’Menouer, Oran, 31000, Algeria; 2 Centre de Recherche Scientifique et Technique en Analyses Physico-chimiques (CRAPC), BP 384-Bou-Ismail-RP, Tipaza, Algeria, 42004, Algeria

**Keywords:** Gallicacid; polyethylene glycol; di-gallate; biopolymer; green synthesis; solid-solid reaction; esterification.

## Abstract

**Background:**

Antioxidant polyphenols like gallic acid (GA) and its esters called “gallates”, which have health advantages for humans, have grown in significance in maintaining a healthy lifestyle and eating a significant amount of secondary plant phytochemicals. Here, for the first time, we suggest a green synthesis of a brand-new, all-natural diester based on gallic acid and polyethylene glycol.

**Methods:**

This di-gallate is created in a single step without the use of a solvent (solid-solid reaction). This reaction has a potential yield of up to 90%. The bathochromic shift of the absorption bands from 277 nm to 295 nm in the UV-VIS spectra was caused by the addition of PEG to gallic acid. To confirm the structure of this di-gallate; Fourier-transform infrared (FTIR) spectroscopy, proton and carbon nuclear magnetic resonance ( ^1^H and ^13^C NMR), the thermal stability identified by thermogravimetric analysis (TGA), X-ray diffraction (XRD), and scanning electron microscopy (SEM) were all used to thoroughly analyze the manufactured product.

**Results and conclusions:**

The acquired results, when compared to the literature spectrums, supported the establishment of the di-ester structure and created new opportunities for a large number of applications.

## Introduction

The introduction “Our food should be our medicine and our medicine should be our food”, Hippocrates' description, though thousands of years old, used to emphasize the importance of nutrition to prevent or cure disease. A sizable group of bioactive phytochemicals known as polyphenols are now receiving more and more attention from the scientific community as well as, most notably, from the general public due to their abundance in foods like fruits, vegetables, and beverages, the regular consumption of which is thought to be good for human health. In addition to their chemopreventive and anticancer potential, studies have demonstrated that polyphenols and their derivatives can reduce the risks of diabetic, cardiovascular, and neurological illnesses. ^1^^,^^2^ Although more recently, the produce, cosmetic and pharmaceutical industries have been using "polyphenols" for their antioxidant properties. According to their chemical structure, polyphenols possess at least two benzene rings and at least one or more hydroxyl substituents characterizing the phenyl system, ^3^ and they are classified into four subclasses, including flavonoids, phenolic acids, stilbenes, and lignans. ^4^

Regarding phenolic acids compounds, are non-flavonoid polyphenolic compounds characterized by a carboxyl group linked to phenyl ring, ^5^ this group includes cinnamic acids (Caffeic, ferulic and p-coumaric acids) and benzoic acids (gallic, vanillic, and syringic acids). When it comes to gallic acid (GA, 3,4,5-tri-hydroxylsbenzoic acid), is the most common hydroxybenzoic acid, that originates from plants and can be produced by acid hydrolysis of hydrolyzable tannins ^6^ or synthesized from shikimic acid, ^7^ these compound present different pharmacological activities, mostly antioxidant activity. The GA alkyl esters (gallates) are an an vital elegance of herbal phenolic compounds that may be extracted from flora or synthesized through esterification of gallic acid with the corresponding alcohol in the presence of the catalyst. ^8^^,^^9^ These alkyl gallates, specifically those with more than seven carbon atoms in the side-chain, have greater favorable and potent activities than gallic acid itself, ^10^^,^^11^ that have precious biological effects, such as anti-microbial, anti-inflammatory, ^12^ antitumor, ^13^ antifungal, ^14^ or prevention of gastrointestinal diseases, diabetes and even cardiovascular diseases. ^15^ In many cases, these GA alkyl esters are useful as food additives ^16^^,^^17^ or beauty additives. ^18^

Due to its high latent heat capacity, ^19^ biocompatibility, solubility in aqueous solutions, and other promising biological properties, polyethylene glycol (PEG) is a synthetic polymer, a polyether made from ethylene glycol, considered as a biopolymer, is widely used in medical and pharmaceutical applications. The strong polarity of PEGs is a result of the terminal hydroxyl and ether groups. ^20^^,^^21^ Because the low molecular weight PEGs have more hydroxyl groups than their structure would suggest, they are less soluble in water and other solvents as their molecular weight rises. PEG is easily chemically altered and can connect to other molecules and surfaces. PEG modifies the solubility and enlarges the connected molecules' size when it is attached to other molecules. ^22^ Polyethylene glycol esters are also used in the medical, cosmetic and food industries. It is made from polyethylene glycol and the corresponding acid.

Here, we present GA/PEG composites which combine the activities of gallic acid and polyethylene glycol « di-gallate of polyethylene glycol », through a rather simple and green esterification process and with progressed properties. The method is based on using only reagents, i.e. solvent-free reaction in a simple Erlenmeyer flasks (“solid-solid reaction”). This method increases the yield of the reaction compared to the standard reaction. The structure of the synthesized di-gallate was confirmed by different analyses: FTIR, ^1^H NMR, ^13^C NMR, UV-Vis, TGA and XRD. We noted the absence of the acid function and the presence of the ester function confirming that the two reagents; gallic acid and polyethylene glycol are associated. The goal of such research work is to develop an efficient and rapid method of the synthesis of GA/PEG composites to be able to acquire di-gallate composites and their changeable properties.

## Methods

### Apparatus

**Fourier Transformed Infrared (FTIR) Spectroscopy;** The FTIR spectra were checked in utilizing a Brucker Tensor-27. The scan was done between 4000 and 400 cm-1. All spectra were baseline-corrected with Opus software.

**X-ray Diffraction (XRD);** The Cristallinity of the products were determined by Brucker D8 Advance diffractometer DAVINCI model, operating in Bragg–Brentano geometry, with Cu Kα radiation (λ = 1.5418 Å).

**Nuclear Magnetic Resonance (NMR);** Proton and carbon nuclear magnetic resonance (1H NMR) and (13C NMR) spectra were recorded on Bruker 300 MHZ NMR spectrometer using deuterated dimethyl sulfoxide (DMSO-d6) as solvent.

**Ultraviolet-Visible (UV–Vis);** UV-Vis absorption spectra were recorded using Evolution 60S spectrophotometer (Thermo Fischer Scientific). The spectra were recorded in Dimethylsulfoxide (DMSO) solvent.

**Scanning Electron Microscopy (SEM);** The analysis of nanocomposites powder image was carried out Quanta FEG 250 instrument (FEI, Hillsboro).

**Thermogravimetric analysis (TGA)**; it was carried out on a Setsys Evolution analyzer (ATG Setaram, Caluire, France) equipped with an aluminium cell, using aluminium pans to encapsulate the samples. Typically, samples were heated at a constant rate of 10 °C/min from room temperature up to 550 °C, under a helium flow of 50 mL/min. The thermal decomposition temperature was taken at the onset of significant (≥5%) weight loss from the heated sample.

### Materials

The gallic acid was purchased from BioChem, Polyethylene glycol glycol 2000 (PEG, M = 2000g/mole) was obtained from Fluka, sulfuric acid was purchased from Emsure and dichloromethane was obtained from Sigma Aldrich.

Synthesis of of polyethylene glycol diester

**Figure 1.**
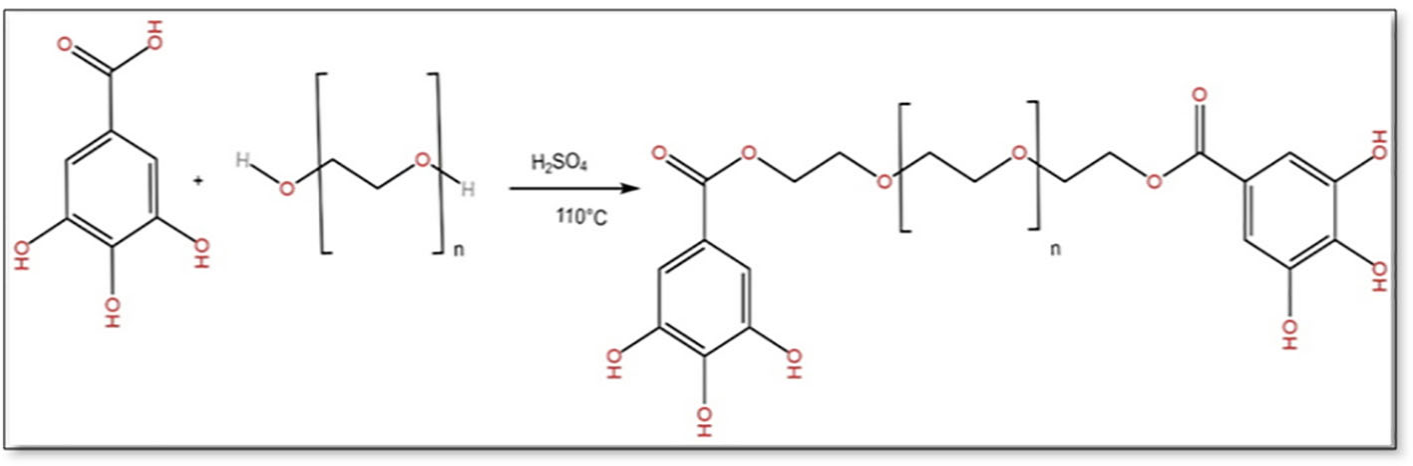
Formation process of Di-gallate.

In this work, we performed a green esterification synthesis using the solid-solid reaction. PEG-digallates was prepared from gallic acid (0.02mol) and polyethylene glycol (PEG) (0.01mol) at 110°C using sulfuric acid (0.5ml) as a catalyst for 12 hours. Thereafter, 50mL of dichloromethane was added and filtred to remove non-reagent amounts of gallic acid and polyethylene glycol. The synthetic scheme is shown in Figure 1.

## Results and discussion

The solid-solid reaction can increase the yield of the esterification reaction until 20% compared with the standard esterification reactions. The final product was analyzed by IR-FT, ^1^H NMR and ^13^C NMR spectroscopy and compared with literature spectra.

### Vibrational characterization by FTIR spectroscopy

**Figure 2.**
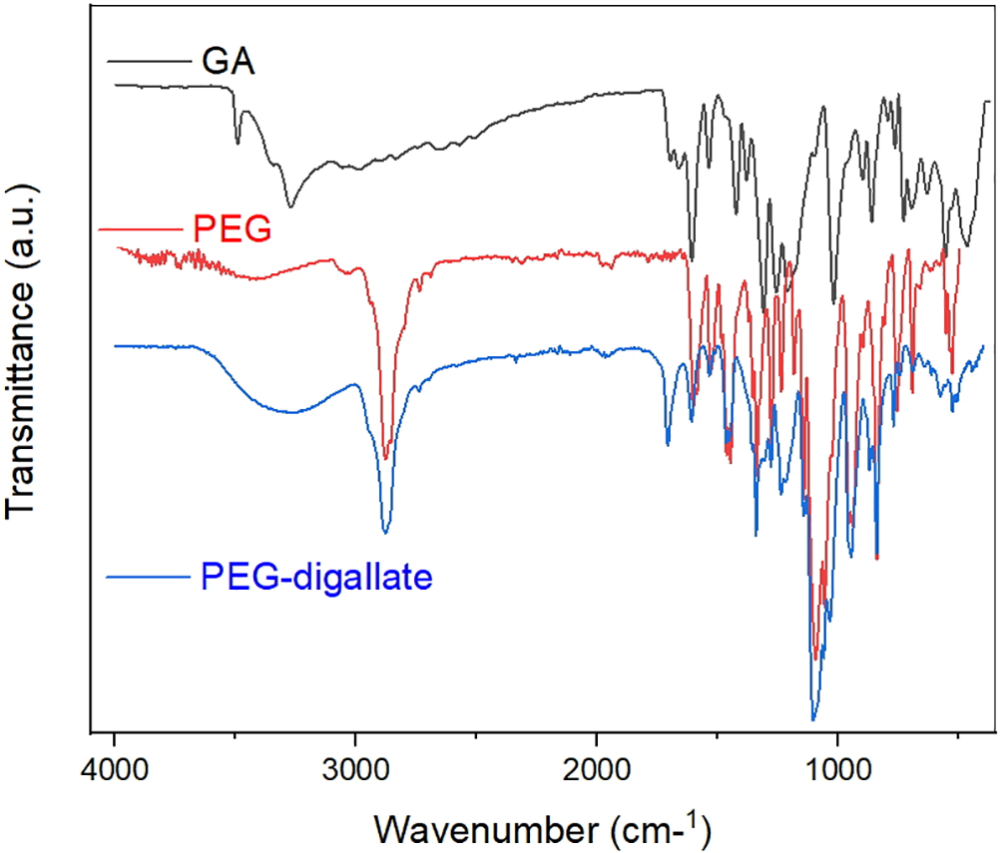
FT-IR spectra of the prepared composite (PEG-digallate), pure PEG and gallic acid.

FTIR analysis was used to identify changes or structure inter¬actions caused by the addition of PEG to Gallic acid. The FT-IR spectra of gallic acid, PEG and PEG-digallate complex treated using Origin Pro (v.2018), are given in Figure 2. As proven in PEG-digallate, the peaks around 3000-3500 cm- ^1^ represented the stretching and bending vibration of -OH, indicating the attendance of hydroxyl groups. The high absorption peaks at 2876, 1707 and 1279 cm- ^1^, were assigned to methylene (=CH _2_), carboxyl (–COOH), and phenol (–OH); groups in pure PEG, respectively; at the same time as the peaks at 1609 and 1104 cm- ^1^ were attributed to aromatic ester and ether groups. ^23^ It is worth noting that, the FT-IR spectra of the obtained complex, pure PEG and GA are relatively similar with several other peaks observed around 1700–1000 cm- ^1^ which are attributed to C–O bond stretching vibration and O–H bond bending vibration into gallic acid. In contrast, changes in the stretching frequency of the current groups indicated the interaction of PEG with the gallic acid functional group.

### NMR analysis

Nuclear magnetic resonance (NMR) spectroscopy was used to determine the molecular structure and chemical composition of the complex PEG-digallate synthesized.

**Figure 3.**
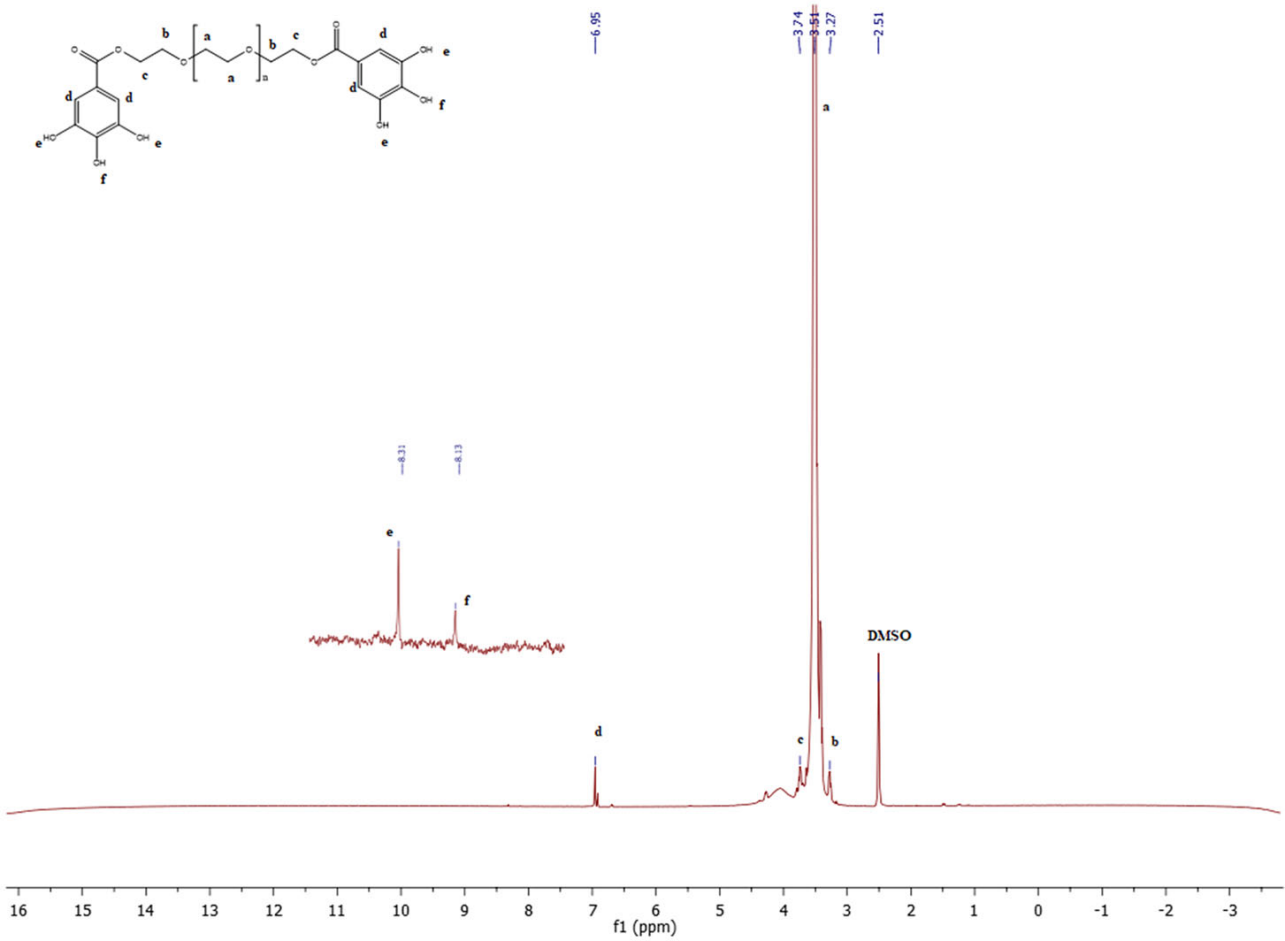
^1^H-NMR spectra of PEG-digallate in DMSO-d _6_.

**Figure 4.**
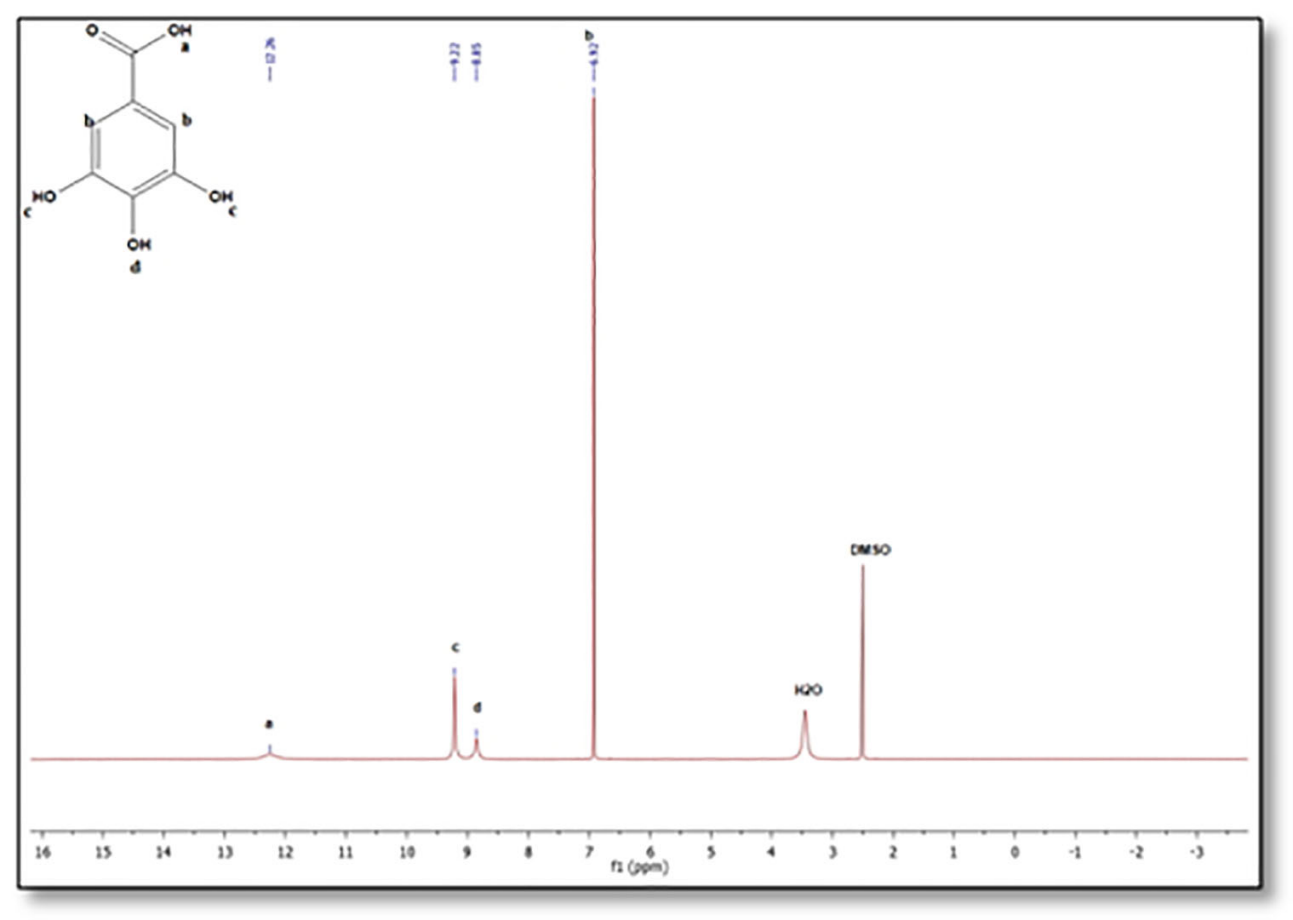
^1^H-NMR spectra of gallic acid in DMSO-d _6_.

The ^1^H-NMR spectra of PEG-digallate and gallic acid have been acquired in DMSO-d6 at a 5 mM concentration. As proven in Figures 3 & 4, the chemical shift of every proton for gallic acid and the obtained product were studied in DMSO-d _6_ earlier than and after reaction with PEG. The signals at 6.95, 8.13, and 8.31 ppm correspond to the ortho protons of the benzene ring, the para and meta hydroxyl (O-H) protons, respectively. Notably, the disappearance of the acidic group (COOH) proton signal and the presence of the polyethylene glycol proton CH2-CH2 are indicated at 3.51 ppm. Additional signals appear at 3.4 and 3.6 ppm, corresponding to protons at the ends of the polymer chains (protons c and b; Figure 3). Comparing the ^1^H-NMR spectra of gallic acid and PEG-digallate, we note that at 12.26 ppm there is no signal corresponding to the -COOH proton of the acid functional group, but there is a signal corresponding to the -OH proton of the hydroxyl functional group at 8.13 and 8.31 ppm, confirming that gallic acid has reacted with the acid function and that the resinous hydroxyl function is free.

Furthermore, comparing the ^1^H NMR spectra of PEG-digallate and pure polyethylene glycol (From literature ^24^), we take a look at the same strong signal at 3.51 ppm, corresponding to the CH _2_-CH _2_ of polyethylene glycol.

The CH _2_-CH _2_ protons at the ends of the polymer chains maintain the same signal at 3.4 and 3.6 ppm.

**Figure 5.**
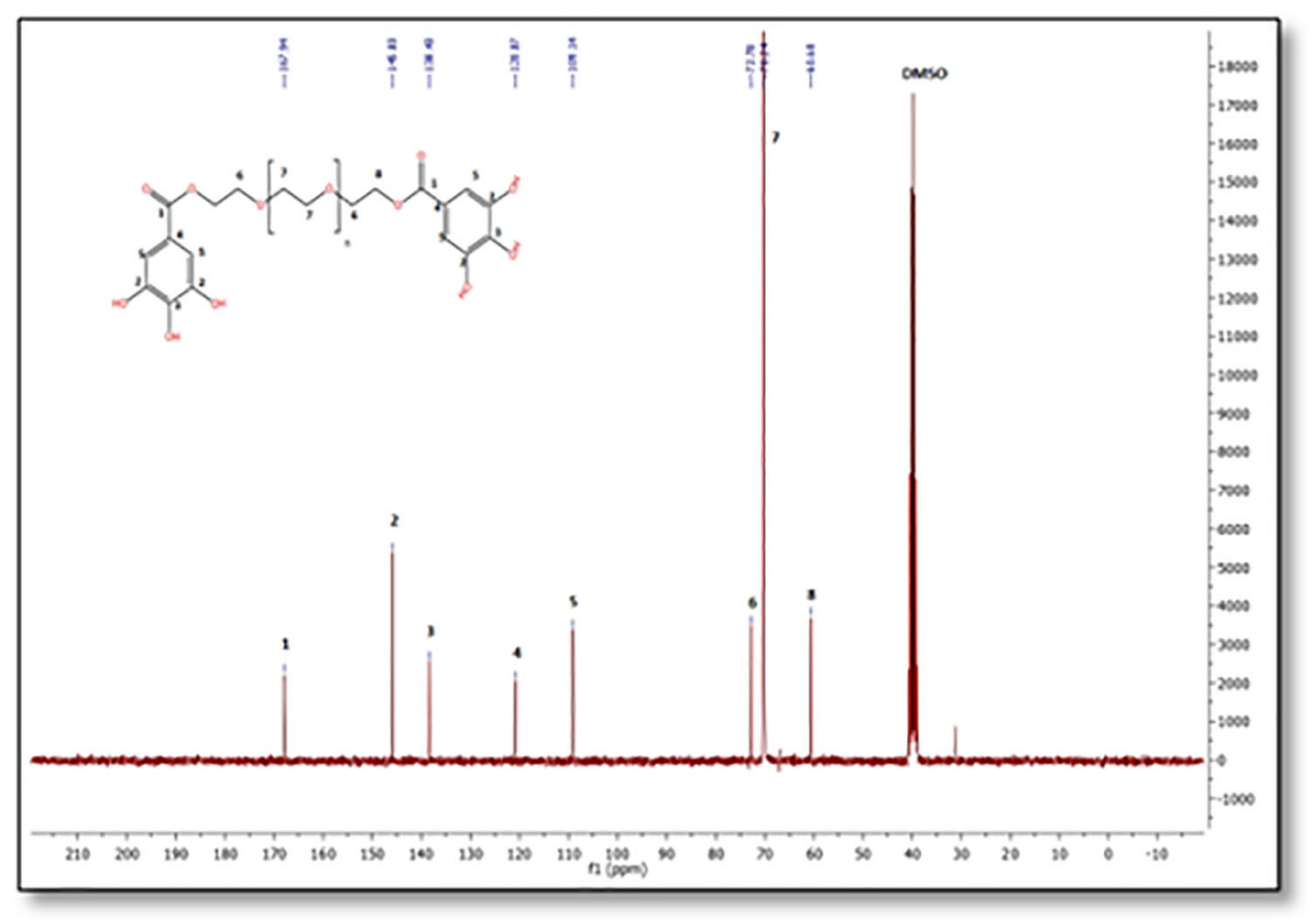
^13^C-NMR spectra of PEG-digallate in DMSO-d _6_.

**Figure 6.**
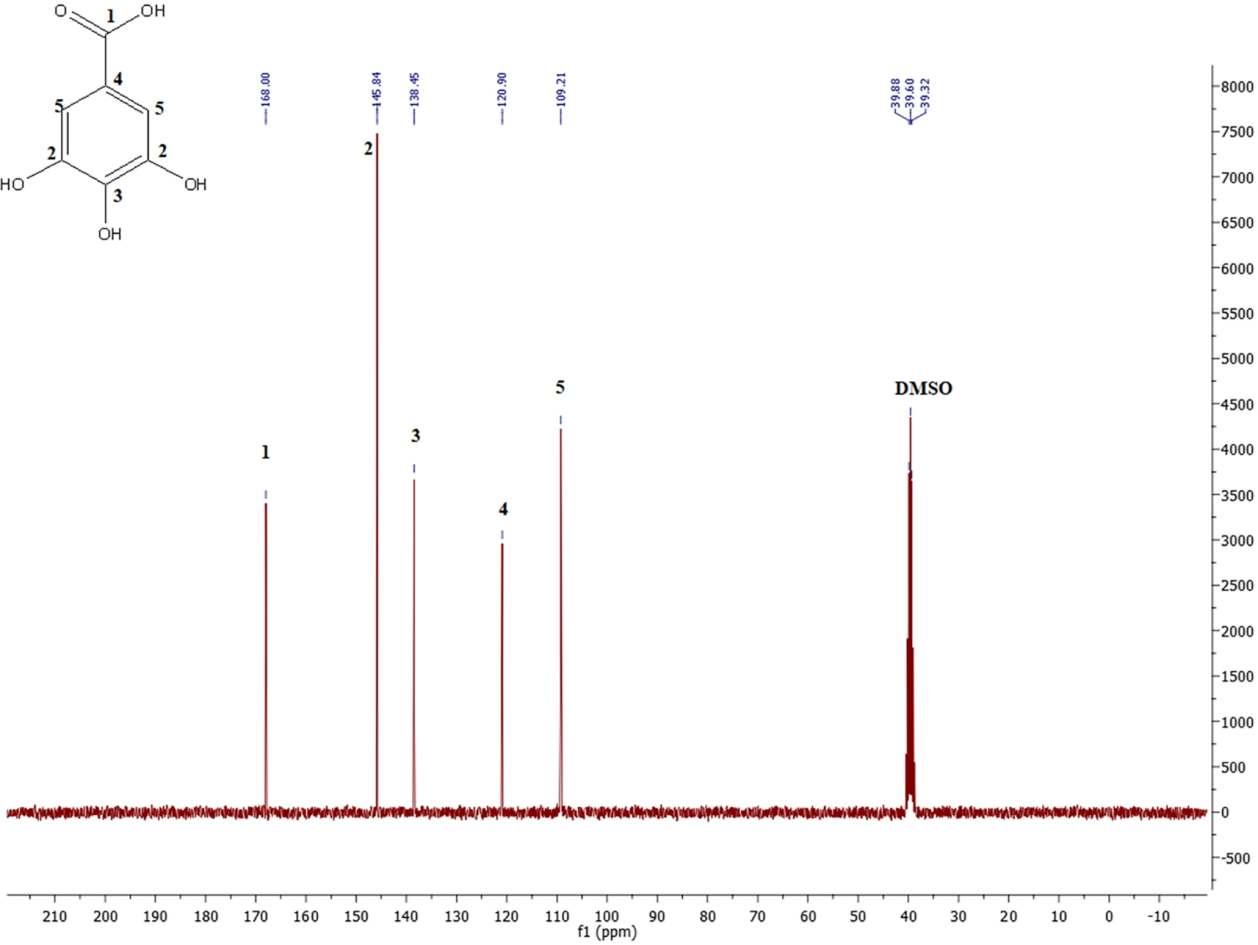
^13^C-NMR spectra of Gallic acid in DMSO-d _6_.

Based at the evaluation of ^13^C-NMR spectra of the obtained complex PEG-digallate and gallic acid ( Figure 5 and 6), which revealed the presence of a carbon signal (O=C-O) at 167.94 ppm corresponds to the ester function. Another peak at 138.4 ppm corresponds to the C-OH carbon in the para position, and a peak at 145.83 ppm corresponds to the two C-OH carbons in the meta position. The signal at 120.87 ppm corresponds to the -C- carbon in the para position, and the two carbons in the ortho position occur at 109.14 ppm on the benzene ring. The strong signal at 70.24 corresponds to the CH _2_-CH _2_ carbons of polyethylene glycol. The signals at 72.78 ppm and 60.68 ppm correspond to the C-O ether and C-O ester carbons at the end of the chain, respectively.

Comparing these results with the literature demonstrates the presence of signals for all carbons corresponding to gallic acid. ^25^ The results in Figure 6 are compared with the ^13^C-NMR spectrum of polyethylene glycol esters, showing the same strong signal at 70.24 ppm, corresponding to the CH _2_-CH _2_ carbons of polyether, and the shift peak of C-O-ester carbons appearing at 60, 68 ppm. ^26^

### X-ray diffraction analysis

**Figure 7.**
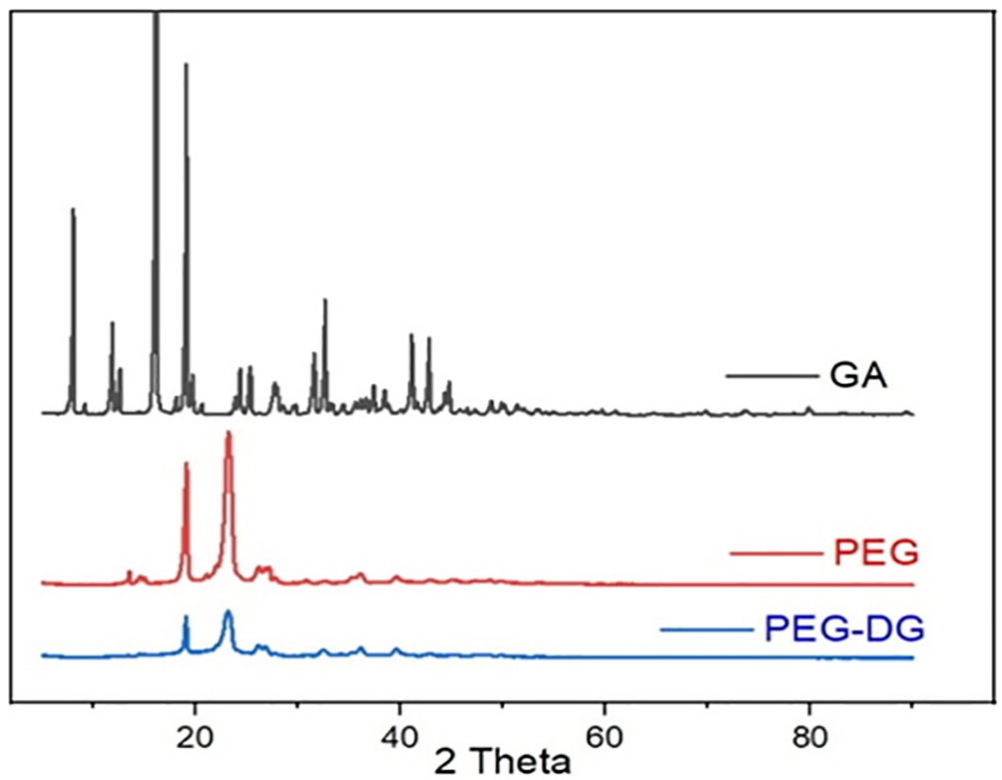
X-ray diffraction patterns of pure gallic acid, pure PEG and the composite PEG-DG.

X-ray diffraction (XRD) analysis was performed to examine the physical state of the obtained PEG-digallate complex. Figure 7 displays the results of the XRD examinations of gallic acid, PEG, and the composite PEG-digallates. Gallic acid was discovered to have numerous strong diffraction peaks of crystallinity between a diffraction angle of 2 θ = 7-50° in the X-ray diffractogram, indicating that it is exists as a crystalline substance. ^27^^,^^28^ Pure PEG displayed two distinct high intensity peaks at approximately 2 θ = 19.20° and 23.20° with a few minor peaks at: 2 θ =26.35, 36.18, 39.84 and 45.29°, respectively. Complex development, where the typical PEG peaks of the composite PEG-DG are present with slightly decreased strength and the elimination of huge diffraction peaks, where it is no longer feasible to detect the characteristic peaks of the composite, both contributed to a partial amorphization. The findings showed that gallic acid is no longer a crystalline substance and that the PEG complex it was successfully formed into still exists in a semi-amorphous condition.

### Scanning electron microscopy

**Figure 8.**
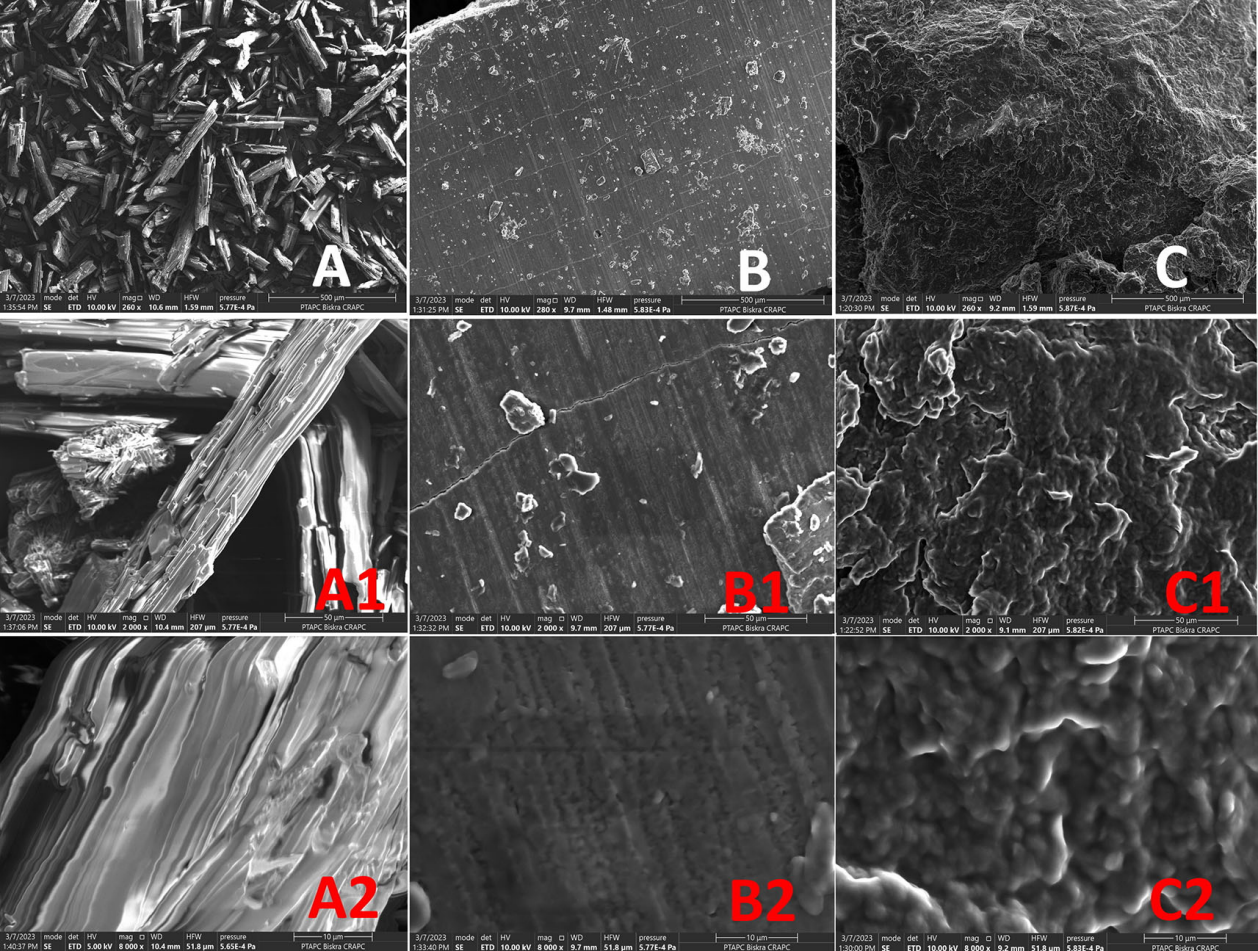
SEM images of GA (A; 500μm, A1; 50μm, A2; 10μm), PEG (B; 500μm, B1; 50μm, B2; 10μm), and PEG-DG (C; 500μm, C1; 50μm, C2; 10μm).

Figure 8 displays SEM images of the complex PEG-Digallate, pure PEG, and gallic acid; they were taken with zooms up to 500, 50, and 10 μm, respectively. Smaller, regular-shaped crystals with a surface that appeared smooth define the pure gallic acid, which also has smaller, more crystalline forms ( Figure 8, A). In contrast to the PEG-digallate composite, pure PEG has an extremely smooth and flat surface and very regular compact structure ( Figure 8, B). Scanning electron micrographs reveal that the surface morphology of the PEG-digallate ( Figure 8, C) composite was amorphous; this may have contributed to the absence of crystal structures (confirming the X-ray diffraction results) and may be related to the uniform dispersion of GA in the PEG matrix.

### UV-Vis absorption measurement

**Figure 9.**
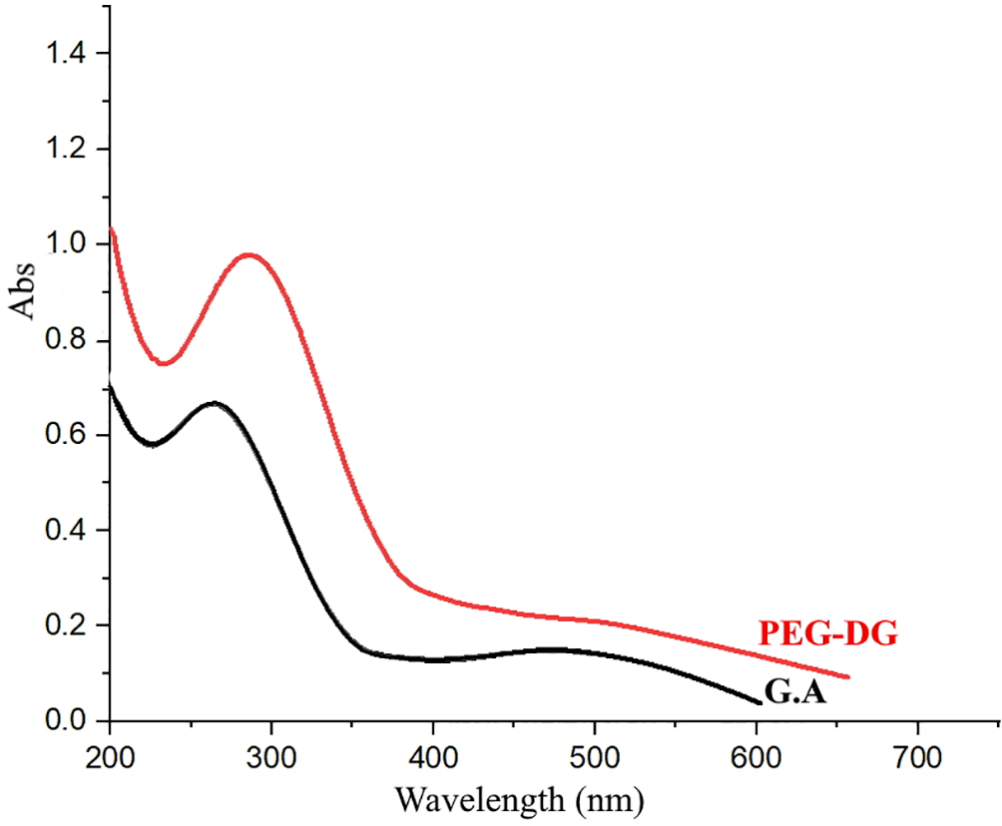
UV-Vis spectra of gallic acid and PEG-digallate in DMSO as solvent.

**Figure 10.**
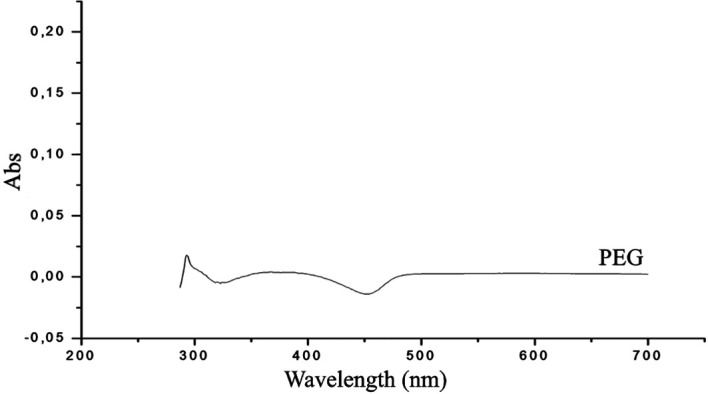
UV-Vis spectrum of polyethylene glycol in DMSO.

The UV-Vis absorption spectra of PEG, GA and PEG-digallate dissolved in DMSO were recorded at room temperature, as shown in Figures 9 & 10.

The absorption spectral changes of PEG-digallate in the presence and absence of PEG are shown in Figure 9. From the obtained spectrum, the absorbance maximum of gallic acid was discovered at 277 nm, which was related to the absorption of aromatic amino acids. The peaks of PEG-digallate increased with the addition of GA, and a red shift at 295 nm was noted. The outcomes suggested that polyethylene glycol and gallic acid had formed a novel combination. A transparent compound in a spectral domain, when taken in an isolated state, can become absorbent if it is put in the presence of a species with which it interacts by a mechanism of the donor-acceptor (D-A) type. According to the UV-Vis absorption spectra of PEG in DMSO, there was no discernible absorption between 200 and 800 nm, demonstrating that pure polyethylene glycol has a relatively low total absorption in the UV region ( Figure 10). Important for electrochromic applications is this observation.

### Thermal analysis (TGA)

**Figure 11.**
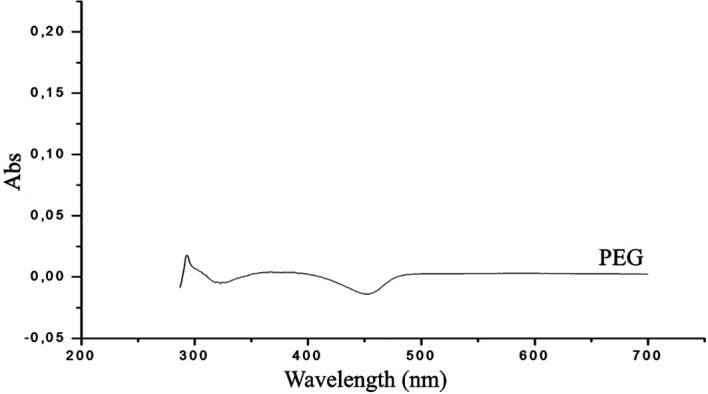
Thermogravimetric analysis TGA of (A) gallic acid, (B) PEG and (C) PEG-digallate.

Thermal gravimetric analysis (TGA) was used to investigate the thermal degradation of PEG-digallate. The TG and DTG curves of PEG, GA, and PEG-digallate were shown in Figure 11 from room temperature to 600 C in a N2 environment at a heating rate of 10°C/min.

The Figure 11 C confirms that the surface medicine was successful. PEG-digallate TG and DTG curves ( Figure 11 C) differ from those of PEG and GA ( Figure 11 A & B). The PEG TG and DTG curves show that PEG has a single breakdown phase that begins at 350 °C and ends about 405 °C. Thermal breakdown of PEG is anticipated to occur at both the backbone chains -C-O- and -C-C- bonds. Figure 11 (A) illustrates that in GA, which has three decomposition phases, the compound is stable up to 87°C when the first mass loss (8.27%) occurs up to 105°C due to hydration water. Gallic acid is stable beyond this temperature until 200°C, with no mass loss and no endo or exothermal processes. The second phase (36.38%) occurs between 220-264°C, and the DTA curve shows an endothermic peak (250°C). The oxidation of organic matter immediately causes the third mass loss (18.22%) and two related exothermic peaks (at 400 and 428°C, respectively). The final residue of gallic acid breakdown was 1.5% of the total original mass (carbon residue). The TG and DTG curves of PEG-digallate in Figure 11C show that the majority of weight loss occurs below 100 °C, which can be attributed to trapped water. Thermal deterioration of composites, on the other hand, occurs primarily around 210 °C and 420 °C, which can be attributed to the GA layer stably coating on PEG. By counting the weight loss from 200 °C to 700 °C, the TG curve reveals that the amount of GA coated on PEG is around 3 to 4 wt.%. Because to the reduction in surface area for alterations, the aggregated PEG-digallate explains the limited amount of coated GA.

## Conclusions

The goal of using a non-solvent and environmentally friendly approach to prepare the specified composite PEG-digallate was discovered to have been achieved. This solid-solid reaction is a synthetic method that maximizes the yield of the reaction (90%) and minimizes waste of chemical reagents. A successful production of PEG-digallate was confirmed by the ^1^H and ^13^C NMR analyses using DMSO-d _6_ as the solvent by the development of new peaks brought on by the interaction of the PEG matrix and gallic acid. The presence of the C-O ether and C-O ester carbons at the end of the chain and hydrogen bonding between the -OH and -COOH groups both appear to play a role in these interactions. Gallic acid absorbs more when PEG is added, with the highest absorption occurring at 295 nm, confirming the UV-Vis spectra of PEG-digallate. We also observed the structure and compact shape of the produced complex, which can support the amorphous composite, by XRD patterns and SEM images. Thermal analysis (TG-DTA) has been shown to be effective approaches for assessing the thermal behaviour of gallic acid. TGA investigated the temperature and enthalpy of melting and solidifying processes to demonstrate the chemical and structural stability of PEG-digllate, which could be maintained even after 200 heat cycles. As a result, this surface modification process paves the way for new applications requiring various types of PEG matrix with gallic acid.

## Data Availability

Figshare: Data di-ester, https://doi.org/10.6084/m9.figshare.23816559.v1. ^29^ Figshare: Figures, https://doi.org/10.6084/m9.figshare.23816619.v1. ^30^ Data are available under the terms of the Creative Commons Attribution 4.0 International license (CC-BY 4.0).
