# Supplementary material for: Green synthesis of new and natural diester based on gallic acid and polyethylene glycol
Source: F1000Res. 2023 Oct 20;12:1384. [Version 1] doi: 10.12688/f1000research.139861.1 (PMC11408915; doi:10.12688/f1000research.139861.1)
Supplement: Peer review history

# Reviewer report: Reviewer response for version 1

**DOI:** 10.5256/f1000research.153173.r261845

**Published:** 2024-09-17

**Authors:** Khadidja Taleb

**Affiliations:** 1 Laboratoire de Synthesè Organique Appliquée (LSOA), Département de chimie, Faculté des sciences exactes et appliquées and Faculté de Médecine, Université Oran 1 Ahmed Ben Bella, Oran, Algeria

Title: Green synthesis of new and natural diester based on gallic acid and polyethylene glycol

 The paper presents the synthesis of a diester based on gallic acid and polyethylene glycol using green method without using solvent (solid-solid reaction).The obtained structure have been characterized using IR spectroscopy, Nuclear Magnetic Resonance ( ^1^H and ^13^C NMR), Thermogravimetric analysis (TGA), X-ray diffraction (XRD) and scanning electron microscopy (SEM). This paper is well structured and the results are interesting and promising for the development of new pathway for di-gallate of polyethylene glycol synthesis. However, Major revision would be required.

 In order to improve the document, the authors are invited to correct some errors in English; some errors of form, which are mentioned below then answer the questions.

  

1. In the abstract (Methods): To confirm the structure of this di-gallate; Fourier-transform infrared (FTIR) spectroscopy, proton and carbon nuclear magnetic resonance (1H and 13C NMR) (something is missing in this sentence  for example “ have been used” )
2. Introduction  (para 2) : The GA alkyl esters (gallates) are an an vital elegance of herbal phenolic
3. Introduction  (para 3): The method is based on using only reagents ( on the use)
4. Methods (Apparatus): The analysis of nanocomposites powder image was carried out Quanta FEG 250 instrument (FEI, Hillsboro) (carried out on)
5. Materials : Synthesis of of polyethylene glycol diester
6. Results and discussion (NMR analysis line 5): earlier than and after. Please use Before instead of earlier
7. Results and discussion (NMR analysis): “we take a look at the same …” in the scientific writing, it is better to avoid first person pronoun, i.e. we etc.
8. Results and discussion (NMR analysis): Based at the evaluation of 13C-NMR. Replace at by on
9. Figure 4 needs to be replaced by a high resolution image.  Peak integration should be displayed in all NMR spectres.
10.  Figure 5 needs to be replaced by a high resolution image
11. I recommend moving Figure 10 to the supporting information (supplementary information).
12. Figure 11. Thermogravimetric analysis TGA of (A) gallic acid, (B) PEG and (C) PEG-digallate (please check this figure and replace it by the adequate figure). Thermogravimetric curves are not presented in this manuscript.
13. In Thermal analysis (TGA) ( line 10 )  and in conclusion :  authors used the abbreviation DTA whereas there is no DTA curves 

Questions

1. In the background: authors have mentioned “Here, for the first time, we suggest a green synthesis of a brand-new, all-natural diester based on gallic acid and polyethylene glycol “in my opinion the diester presented here is not all- natural because it is synthetized by a synthetic polymer PEG and Gallic acid (natural compound) , furthermore the synthetic method des not respect the green chemistry rules (high temperature,toxic concentrated acid) I recommend to change this statement.
2. Introduction (para 3): “This method increases the yield of the reaction compared to the standard reaction”. Could you provide one or two references?
3. In the last paragraph of the introduction “Here, we present GA/PEG composites” this macromolecule is not a composite “see the definition of composite”.   Please correct this in the whole paper.

1. Introduction : para 3 : *“ Here, we present GA/PEG composites which combine the activities of gallic acid and polyethylene glycol « di-gallate of polyethylene glycol »”*  what authors mean by activities ? Do you mean synergetic effect, if it is the case please correct the sentence?
2. Could the authors justify the choice of reaction temperature?

1. No need to include  NMR spectra of gallic acid because it is available in many works  ( example: DOI:10.1371/journal.pone.0140242
2. Figure 11. Thermogravimetric analysis TGA of (A) gallic acid, (B) PEG and (C) PEG-digallate  ( please check this figure and replace it by the adequate figure). There is no Thermogravimetric analysis TGA in this manuscript.
3. In Thermal analysis (TGA) (line 10):  authors used the abbreviation DTA; meanwhile there is no DTA curves.
4. English of the paper should be corrected professionally.
5. Please provide more details about potential applications of this macromolecule.

Is the work clearly and accurately presented and does it cite the current literature?

Yes

If applicable, is the statistical analysis and its interpretation appropriate?

Not applicable

Are all the source data underlying the results available to ensure full reproducibility?

Yes

Is the study design appropriate and is the work technically sound?

Yes

Are the conclusions drawn adequately supported by the results?

Yes

Are sufficient details of methods and analysis provided to allow replication by others?

Partly

Reviewer Expertise:

The whole article have been read, checked  and experted

I confirm that I have read this submission and believe that I have an appropriate level of expertise to confirm that it is of an acceptable scientific standard, however I have significant reservations, as outlined above.

# Reviewer report: Reviewer response for version 1

**DOI:** 10.5256/f1000research.153173.r243716

**Published:** 2024-09-12

**Authors:** Azhagu Madhavan Sivalingam

**Affiliations:** 1 Natural Products & Nanobiotechnology Research Lab, Department of Community Medicine, Saveetha Medical College, Saveetha Institute of Medical and Technical Sciences, Chennai, Tamil Nadu, India

1. The abstract does not sufficiently explain the rationale behind in this study

 2. The Introduction contains several disjointed statements that make it difficult to follow the main purpose of the study, and clarity of these sentences.

 3. The methodology mentions the use of analytical equipment and providing specific models or manufacturing and specificity makes.

 4. The whole phytochemical compound benefits or evaluates the significance of the findings.

 5. The conclusion does not elaborate on the potential mechanism.

Is the work clearly and accurately presented and does it cite the current literature?

Yes

If applicable, is the statistical analysis and its interpretation appropriate?

Yes

Are all the source data underlying the results available to ensure full reproducibility?

Yes

Is the study design appropriate and is the work technically sound?

No

Are the conclusions drawn adequately supported by the results?

Yes

Are sufficient details of methods and analysis provided to allow replication by others?

Yes

Reviewer Expertise:

Plant based nanotechnology, Pharmacology,

I confirm that I have read this submission and believe that I have an appropriate level of expertise to confirm that it is of an acceptable scientific standard.

## References

[1] : Polyphenol-compounds From Green Synthesis of Antimicrobial property of Silver Nanoparticles using Eichhornia crassipes: Characterization and Applications. *Silicon* .2023;15(17) : 10.1007/s12633-023-02593-2 7415-7429 10.1007/s12633-023-02593-2

[2] : Green synthesis and characterization of silver nanoparticles with different solvent extracts of Sesbania grandiflora (L.) Poiret and assessment of their antibacterial and antioxidant potentials. *Biomass Conversion and Biorefinery* .2023; 10.1007/s13399-023-04696-7 10.1007/s13399-023-04696-7

# Reviewer report: Reviewer response for version 1

**DOI:** 10.5256/f1000research.153173.r261846

**Published:** 2024-04-30

**Authors:** Nur Izyan binti Wan Azlee

**Affiliations:** 1 Institute of Bioproduct Development (IBD), University of Technology Malaysia, Skudai, Johor, Malaysia

**General comments:**

1. The manuscript contains several grammatical and technical errors.
2. The highlighted sentences require rephrasing/corrections/changes/clarifications.
3. The discussion part seems to be lacking in contrast with the abundance of results obtained.

**Specific comments:**

1. What is the concentration of sulfuric acid catalyst used for the synthesis of polyethylene glycol diester in the study? The mentioned volume of 0.5ml is for what total volume? Needs further clarification.
2. Suggest mentioning the ratio of GA/PEG synthesized in the study.
3. Suggest improving the resolutions for Figure 1, Figure 4, Figure 5.
4. Organize all figures to be after the mentioned text.
5. Change the sub-titles in the Results and Discussion to be in terms of the result from the analyses and not the name of the analysis itself. Example: ‘Scanning electron microscopy’ to be changed to ‘Electron micrographs of the samples’.
6. Figure 8: Insert the magnifications for each image.
7. At the conclusion section, the authors mentioned ‘TGA investigated the temperature and enthalpy of melting and solidifying processes to demonstrate the chemical and structural stability of PEG-digllate, which could be maintained even after 200 heat cycles.’ Is there any evidence for the claimed 200 heat cycles done?
8. It is suggested for the authors to strengthen the discussion section by comparing the data analysis obtained from the study with previous studies reported earlier.
9. Specify the methodology further.

Please also see the attached file linked here containing additional review comments.

Is the work clearly and accurately presented and does it cite the current literature?

Partly

If applicable, is the statistical analysis and its interpretation appropriate?

Yes

Are all the source data underlying the results available to ensure full reproducibility?

Partly

Is the study design appropriate and is the work technically sound?

Yes

Are the conclusions drawn adequately supported by the results?

Partly

Are sufficient details of methods and analysis provided to allow replication by others?

Partly

Reviewer Expertise:

Enzyme technology, bioprocessing, bioconversion, biomass degradation

I confirm that I have read this submission and believe that I have an appropriate level of expertise to confirm that it is of an acceptable scientific standard, however I have significant reservations, as outlined above.

# Response

**Published:** 2024-05-22

**Authors:** Fatima zohra Zeggai

**Affiliations:** Chemistry, Not applicable, Oran, Algeria

Thank you, We will do our best to correct the manuscript

 Kind regards
